# Evaluation of Cow’s Milk Related Symptom Score [CoMiSS] accuracy in cow’s milk allergy diagnosis

**DOI:** 10.1038/s41390-023-02539-9

**Published:** 2023-03-04

**Authors:** Ali M. El-Shafie, Zein A. Omar, Heba M. S. El Zefzaf, Elsayedamr M. Basma, Nahla M. Al Sabbagh, Wael A. Bahbah

**Affiliations:** 1 Department of Pediatrics, Faculty of Medicine, Menoufia University, Shebin El-Kom, Egypt; 2 Department of Bioinformatics and Medical Statistics, Medical Research Institute, Alexandria University, Alexandria, Egypt

## Abstract

**Background:**

Cow’s Milk-related Symptom Score (CoMiSS) is an awareness tool to recognize cow’s milk allergy (CMA) symptoms in infants. We aimed to assess the best cut-off point of CoMiSS in our country and investigate other parameters suggested to raise the strength of CoMiSS in CMA diagnosis.

**Methods:**

We enrolled 100 infants with CMA-suggestive symptoms with documentation of CoMiSS initially and 4 weeks after cow milk-free diet (CMFD) followed by an open food challenge (OFC) test. Infants with symptom recurrence upon challenge were diagnosed with confirmed CMA.

**Results:**

Initial mean CoMiSS was 15.76 ± 5.29, being higher in the confirmed CMA group (84% of infants). Following CMFD, median CoMiSS significantly reduced to 1.5 in the confirmed CMA group compared to 6.5 in the negative group. Receiver operation characteristic (ROC) curve identified a CoMiSS score of ≥12 as the best cut-off value with 76.19% sensitivity, 62.50% specificity and overall accuracy of 74.00%. Mucoid stool, bloody stool and faltering growth were reported in 80, 41 and 52% of confirmed CMA infants, respectively, with considerable improvement following CMFD.

**Conclusions:**

Our study revealed a CoMiSS score of ≥12 to be the best cut-off point. However, CoMiSS cannot be used alone for accurate diagnosis of CMA.

**Impact:**

- CoMiSS *≥*12 can predict a positive response to CMFD; nevertheless, CoMiSS is a good awareness tool and cannot be regarded as a stand-alone CMA diagnostic test.
- CoMiSS reduction following CMFD was predictive of a reaction to OFC to diagnose CMA as well as for monitoring symptom improvement.
- Symptoms commonly associated with CMA as mucoid stool, bloody stool, marked abdominal distention not responding to medical treatment and faltering growth, in addition to their improvements in response to CMA are suggested parameters to be added to CoMiSS to improve its accuracy.

## Introduction

Food allergy rates have a wide range of variability by age, diet, and many other factors, however eight types of food account for more than 90% of allergic reactions in affected individuals: milk, eggs, fish, tree nuts, peanuts, soy, shellfish, and wheat.^1^ Cow’s milk allergy (CMA) is the abnormal immune response to proteins found in cow milk or its products. The reported prevalence of CMA is less than 5.0%, and according to the EuroPrevall data, the prevalence of CMA is even as low as 0.54%.^2^ Cow’s milk has more than 20 protein fractions, the most significant allergens are casein protein that includes alpha-s1, alpha-s2, beta, and kappa casein and also whey proteins that include alpha-lactalbumin and beta-lactoglobulin.^3^ CMA reactions have an immunological base and are classified into IgE mediated, non-IgE mediated and mixed types.^4,5^ Most IgE-mediated reactions usually involve the skin, but most of the non-IgE-mediated reactions involve the gastrointestinal tract. In IgE-mediated food hypersensitivity, infants develop atopic dermatitis, urticaria, diarrhea, vomiting, shortness of breath, laryngeal edema, and/or hypotension with cardiovascular collapse and anaphylaxis. Non-IgE-mediated present with diarrhea, emesis, and if prolonged faltering growth and bloody stool.^6^ Some symptoms of CMA such as diarrhea, constipation, regurgitation, and colic are common in functional GI disorders of infants. As there is no gold-standard diagnostic test for CMA and the diagnosis depends on an Open Food Challenge (OFC) test, so differentiation between functional GI disorders and CMA in infants presenting with these overlapping symptoms is usually difficult.^7^

So developing an awareness tool was essential to help in the identification of infants suspected to have CMA, particularly those with non-IgE mediated types.^8^ This occurred in September 2014 in Brussels, Belgium with the development of Cow’s Milk-related Symptom Score (CoMiSS). Crying, regurgitation, stool pattern, skin, and respiratory symptoms were the domains defined for the CoMiSS score, and a cut-off point of 12 was recommended in this consensus as a potential score to indicate CMA. The score was assessed in infants with suspected symptoms at the initial diagnosis of CMA and when OFC was done it was positive in 80% of infants whose CoMiSS score decreased after 4 weeks of elimination to ≤6.^8^ But it was recently suggested that this cut off point be lowered from ≥12 to ≥10 with replacement of the Bristol Stool Scale (BSS) by the Brussels Infant and Toddlers Stool Scale (BITSS) as it is more specific for non-toilet trained infants; however, this did not change the impact of stool characteristics on CoMiSS.^9^ Despite being a clinically useful tool in identifying infants with CMA, CoMiSS cannot be used as a cornerstone for diagnosis of infants with obvious CMA such as those with anaphylactic or immediate IgE-mediated reactions who will not be picked up by the CoMiSS, but it may be used as a helpful tool to identify infants with persisting GI symptoms who may benefit from cow milk-free diet (CMFD) when the diagnosis of CMA is suspected.^10,11^

So, we aimed in this study to assess the best CoMiSS cut off point predictive of response to CMFD and to investigate other parameters suggested to raise the strength of CoMiSS as a useful tool in CMA diagnosis.

## Methods

### Design of the study

This prospective study was conducted to assess CoMiSS accuracy in CMA diagnosis in infants presenting with acute or chronic symptoms suggestive of CMA. CoMiSS is a simple awareness tool rating five symptoms: daily duration of crying, number and volume of episodes of regurgitation, consistency of stools, presence and severity of atopic eczema or urticaria, and presence and severity of respiratory symptoms with a total score ranging from 0 to 33 points (Table 1). Crying, regurgitation, and skin manifestations had a score from 0 to 6, according to the severity with each increase of 1 point meaning more severe symptoms up to 6 points for the worst symptom. Stool consistency is scored based on the Bristol stool scale (BSS) as 0 for normal stools (types 3 and 4), 2 for soft stools (type 5), 4 for hard stools (types 1 and 2) or liquid stools (type 6) and 6 points for watery stools (type 7). The respiratory symptoms had a score from 0 to 3, with 0 for no symptoms, 1 for mild, 2 for moderate and 3 for severe respiratory symptoms. Infants presenting with other manifestations suggestive of CMA and not included in CoMiSS were also included in our study as blood in stool, mucoid stool, severe abdominal distention not responding to regular treatment, hematemesis, and failure to gain weight.

**Table 1:** CoMiSS score.

Symptom	Score			
Crying (only considered if the child has been crying for 1 week or more assessed by parents)	0	≤1 h/day		
	1	1–1.5 h/day		
	2	1.5–2 h/day		
	3	2–3 h/day		
	4	3–4 h/day		
	5	4–5 h/day		
	6	≥5 h/day		
Regurgitation	0	0–2 episodes/day		
	1	≥3–≤5 of small volumes		
	2	>5 episodes of >1 coffee spoon		
	3	>5 episodes of ±half of the feeds in half of the feeds		
	4	Continuous regurgitations of small volumes >30 min after each feed		
	5	Regurgitation of half to complete volume of a feed in at least half of the feeds		
	6	Regurgitation of the complete feed after each feeding		
Stools (Bristol scale)	4	Types 1 and 2 (hard stools)		
	0	Types 3 and 4 (normal stools)		
	2	Type 5 (soft stools)		
	4	Type 6 (liquid stools, if unrelated to infection)		
	6	Type 7 (watery stools)		
		Atopic eczema head–neck–trunk arms–hands–legs– feet		
Skin	0–6	Absent	0	0
		Mild	1	1
		Moderate	2	2
		Severe	3	3
Urticaria	0 or 6	YES	NO	
		6	0	
Respiratory symptoms	0		No respiratory symptoms	
	1		Slight symptoms	
	2		Mild symptoms	
	3		Severe symptoms	

Our primary outcome was to assess the accuracy of CoMiSS compared to OFC for CMA diagnosis and to identify the best CoMiSS cut off point predictive of response to CMFD in order to better identify “which” infants may benefit by CMFD. The secondary outcome was to investigate other presenting symptoms for CMA to be suggested to raise the strength of CoMiSS as a useful tool in CMA diagnosis.

### Study population

We initially included 150 infants who presented to our pediatric gastrointestinal and clinical nutrition clinic in Menoufia University Hospital from January 2020 to May 2021. Out of 150, 20 infants were excluded because their parents refused to participate in the study. We started the elimination diet in 130 infants. Infants whose mothers were non-adherent to CMFD, refused OFC or lost during follow-up (n = 30) were excluded. A total of 100 infants completed the study after a written consent from their parents. The study involving human participants was reviewed and approved by the Institutional Review Boards (IRB) of the Menoufia Faculty of Medicine (ID number: 191019 PEDI 28).

Our inclusion criteria were infants less than one year with persistent unexplained symptoms suspecting CMA such as regurgitation, vomiting, diarrhea, constipation and/or other symptoms and signs such as faltering growth, skin manifestations, sleeping problems, bloody stool, mucoid stool, severe abdominal distention not responding to regular treatment, hematemesis, skin allergy and recurrent unexplained wheezy chest. Infants were excluded if they were older than 12 months of age, had anaphylaxis following CM, receiving extensively hydrolyzed Formula (ehF) or Amino Acid-based Formula (AAF) on initial presentation, had multiple food allergy, had a surgical intervention recently or had gastrointestinal malformations.

Infants were included to confirm or rule out CMA if other explanations of the presenting symptoms were ruled out and symptoms persisted following a trial of symptomatic therapy. For example, behavior modification and nutritional guidance for ensuring adequate intake and thickening formulas (by cornstarch or use of anti-regurgitation formula) in the case of regurgitating infants, excluding ear infections in cases of persistent crying, non-pharmacological (burping during and after feeding, avoidance of over feeding, bicycling motion and gentle massage) and pharmacological treatment (simethicone) of abdominal distention and ensuring adequate nutritional intake in cases of faltering growth in order to prevent overdiagnosis. In presence of bloody stool, we excluded any cause of rectal bleeding (bleeding disorders). In infants with anal fissure, if associated with constipation following switching from breastmilk to artificial formula or introduction of solid foods, they received a trial of treatment for constipation first, if no response they were included in the study. However, those presenting with anal fissure associated with perianal dermatitis, mucoid, and /or bloody stool or other atopic manifestations were included from the start. Then we took complete history (age, sex, type of feeding, family history of allergy, previous formula, and previous hospital admission) and we documented all presenting symptoms included in the CoMiSS score as well as those not included in the CoMiSS score as mucoid stool, bloody stool, hematemesis, and persistent abdominal distention. Complete physical examination was done with an emphasis on anthropometric measures according to *Z* score growth references for Egyptian children from birth up to 5 years,^12^ then cow milk-specific IgE was tested (cow milk-specific IgE ≥0.35 kU/L was considered positive). CoMiSS score was assessed for each case then we started an elimination diet (CMFD) for 4 weeks then OFC was done.

### CoMiSS evaluation

CoMiSS score was completed at the initial visit (T0) [the CoMiSS tool was downloaded from the dedicated website^13^] without limiting cut-off point for inclusion in our study. CMFD was introduced for 4 weeks, and the score was completed by the same clinician (T1). We compared the CoMiSS score of CMA confirmed cases and CMA negative cases (based on result of OFC). We also compared variation of the score in response to CMFD in the form of decrease by 50% from T0 between the two groups.

### Cow milk-free diet

In breast-fed infants, breastfeeding was continued while their mothers avoided all cow milk products from their diet (maternal CMFD). In formula-fed infants, AAF was used (we prescribed AAF as it was cheaper and more available for the infants than extensively hydrolyzed formula in our country).

### Open Food Challenge

OFC is the cornerstone for the diagnosis of CMA according to the 2012 ESPGHAN GI Committee Practical Guidelines. All infants underwent an open oral challenge test performed in our Pediatric Gastroenterology clinic under medical observation according to the guidelines.^14^ Before OFC, infants were examined in detail, emphasizing the cutaneous, respiratory, and gastrointestinal systems. Also, we ensured that infants were free from fever, signs or symptoms of acute infections, runny nose, cough or wheezing. Then very small amounts of Cow Milk (CM) (standard formula) were started with increment gradually as follows 3, 10, 30, 100 up to 250 ml at intervals of 20 minutes. Before each dose administration, infants were examined. Challenge was interrupted when objective signs and symptoms indicated a positive response. After the last dose, Infants were observed for 2 hours to monitor any acute adverse reaction. The manifestations considered related to CMA were: urticaria, rash, pruritus, repeated vomiting, sneezing, rubbing of nose and/or eyes, watery eyes, coughing, wheezing, or frequent diarrhea.

In all, 42.85% of infants in confirmed CMA group (36/84) had acute reactions (defined as reactions occurring during or within 2 hours of the last dose of CM during the challenge). respiratory symptoms were the most common (sneezing, coughing, and wheezing) followed by Cutaneous (urticaria, rash and pruritus) then gastrointestinal (repeated vomiting and diarrhea). Cardiovascular and laryngeal symptoms were not reported in our study. This high percentage can be justified that 25% of infants in confirmed CMA group had positive cow milk specific IgE suggesting IgE-mediated CMA and there was a significant agreement between the reactions reported by the family history and those observed during the challenge.

Parents were instructed to give the infants at least 250 ml per day of a standard CM based formula at home for 14 days. During this period parents continued to monitor symptom recurrence and were instructed to notify the researcher if any delayed reactions occurred and to bring the infant to hospital for reassessment. A positive OFC was considered when symptoms recurred, and the diagnosis of CMA was confirmed. Infants were diagnosed as CMA negative when symptoms did not reappear within 4 weeks.

### Statistical methodology

Demographic data, CoMiSS score, symptoms not included in CoMiSS score, results of the allergy test and of the OFC were all recorded in an Excel database. An independent statistician performed the statistical analysis by using SPSS (Statistical Package for Social Science) program for statistical analysis (ver 25). We used median (minimum–maximum) and inter-quartile ranges (IQR) or mean ± SD for the description of the data. Median values were considered whenever a non-normal distribution was present. We compared the CoMiSS median score at enrollment (T0) and following CMFD (T1). Comparisons were carried out between two studied independent not-normally distributed subgroups using Mann–Whitney *U*-test. Chi-square test was used to test association between qualitative variables. Monte Carlo corrections was carried out when indicated (*n* × *m* table and >25% of expected cells were <5). Significance was set at *p* value <0.05. We calculated the sensitivity, specificity, positive (PPV) and negative predictive value (NPV), and area under the curve (AUC) [receiver operation characteristic (ROC)] by using MedCalc Software version 14. Youden index was used to determine the best cut-off value. We tested the significance and the percentage of improvement of manifestations other than CoMiSS according to their presence or absence. An alpha level was set to 5% with a significance level of 95%.

The minimal sample size was calculated depending on a previous study that aimed to assess the accuracy of CoMiSS in response to CMFD.^11^ Based on their findings and anticipating the ratio of negative/positive infants CMA to be 1:4, a sample size of 18 children (with a minimum sample size for CMA positive infants of 14 children) is the minimum required sample to conduct this diagnostic test accuracy study.^15^ But we narrowed the effect size of our primary outcome (diagnostic accuracy of CoMiSS score) to be 0.6 to yield a larger sample size which was found to be 93 infants (with a minimum sample size for CMA-positive infants of 74 children) is the minimum required sample size to detect a discrimination power of 70% of CoMiSS score for infants having CMA with a level of significance 5% (*α* error accepted 0.05) and statistical power (1 − *β*) of 80%. The sample size increased to 100 infants to control for attrition (withdrawal) bias.^16^

## Results

Out of 100 infants who completed the study (median age was 3.25 months, range 2.00–7.00 months), 52% were underweight, 19% had positive family history of allergy, 21% were exclusively breastfed and 57% were receiving specialized formula at recruitment. 84 infants (84%) were positive for OFC (confirmed CMA) and 25% (21/84) of confirmed CMA group had a positive cow milk specific IgE allergy test. Most of the infants had gastrointestinal complaints, 25% presented with GI manifestations alone, 24% presented with GI and skin manifestations,13% presented with GI and respiratory manifestations, 36% presented with GI, respiratory and skin manifestations and 2% presented with general manifestations (crying and irritability). The mean CoMiSS score of the infants at presentation (T0) was 15.76 ± 5.29 (range, 2–26). Patient characteristics are listed in Table 2.

**Table 2:** Clinical and laboratory data at presentation (T0).

	All patients (*n* = 100)	Negative CMA (*n* = 16)	Confirmed CMA (*n* = 84)	Test of significance, *p* value
Age in months				
Median [min–max]	3.25 [2.00–7.00]	2.50 [1.75–4.00]	4.00 [2.00–7.00]	0.160 NS
Sex				
Male	50 (50.00%)	7 (43.75%)	43 (51.19%)	0.585 NS
Female	50 (50.00%)	9 (56.25%)	41 (48.81%)	
Type of feeding				
Exclusive breast feeding	21 (21.00%)	3 (18.75%)	18 (21.43%)	0.970 NS
Exclusive artificial feeding	43 (43.00%)	7 (43.75%)	36 42.86%)	
Mixed	36 (36.00%)	6 (37.50%)	30 (35.71%)	
Family history of allergy	19 (19.00%)	2 (12.50%)	17 (20.24%)	0.471 NS
Weight for age *Z* score				
Normal	48 (48.00%)	6 (37.50%)	42 50.00%)	
Under weight	52 (52.00%)	10 (62.50%)	42 (50.00%)	0.357 NS
Length for age *Z* score				
Normal	74 (74.00%)	11 (68.75%)	63 75.00%)	
Stunted	26 (26.00%)	5 (31.25%)	21 (25.00%)	0.603 NS
Weight for length				
Normal	72 (72.00%)	10 (62.50%)	62 73.81%)	
Wasted	28 (28.00%)	6 (37.50%)	22 (26.19%)	0.357 NS
Usage of previous specialized formulas^a^	57 (57.00%)	6 (37.50%)	51 (60.71%)	0.085 NS
Lactose-free formula (LF)	33/57 (57.89%)	4/6 (66.67%)	29/51 (56.86%)	0.582 NS
Anti-regurgitation (AR) formula	3/57 (5.26%)	0/6 (0.00%)	3/51 (5.88%)	0.541 NS
Soy-based formula	12/57 (21.05%)	1/6 (16.67%)	11/51 (21.57%)	0.779 NS
Partially hydrolyzed formula (HA)	16/57 (28.07%)	1/6 (16.67%)	15/51 (29.41%)	0.509 NS
Positive specific IgE for cow milk	21 (21.00%)	0 (0.00%)	21 (25.00%)	0.024*
System(s) involved				
General	2 (2.00%)	1 (6.25%)	1 (1.19%)	0.186 NS
GIT only	25 (25.00%)	6 (37.50%)	19 (22.62%)	0.207 NS
GIT and skin	24 (24.00%)	2 (12.50%)	22 (26.19%)	0.238 NS
GIT and respiratory	13 (13.00%)	3 (18.75%)	10 (11.90%)	0.453 NS
GIT, respiratory, and skin	36 (36.00%)	4 (25.00%)	32 (38.10%)	0.317 NS
Total CoMiSS Score (T0)				
Mean ± SD	15.76 ± 5.29	12.44 ± 5.01	16.39 ± 5.13	0.006*
Min–Max	2.00–26.00	6.00–22.00	2.00–26.00	

Normal weight = weight for age between −2 and +1 standard deviation SD on Egyptian *Z* score and underweight = weight for age < −2 SD.

Normal length = length for age between −2 and +3 SD on Egyptian *Z* score and stunted = length for age < −2 SD.

Normal weight for length = weight for length between −2 and +1 SD on Egyptian *Z* score and wasted = weight for length < −2 SD.

*SD* standard deviation.

*Statistically significant (*p* < 0.05); NS: statistically not significant (*p* ≥ 0.05).

^a^The use of previously specialized formulas is NOT necessarily exclusive (a child may use one or more types of formulas; hence the overall percentage may be higher than 100%).

### Clinical presentation

**Fig. 1:**
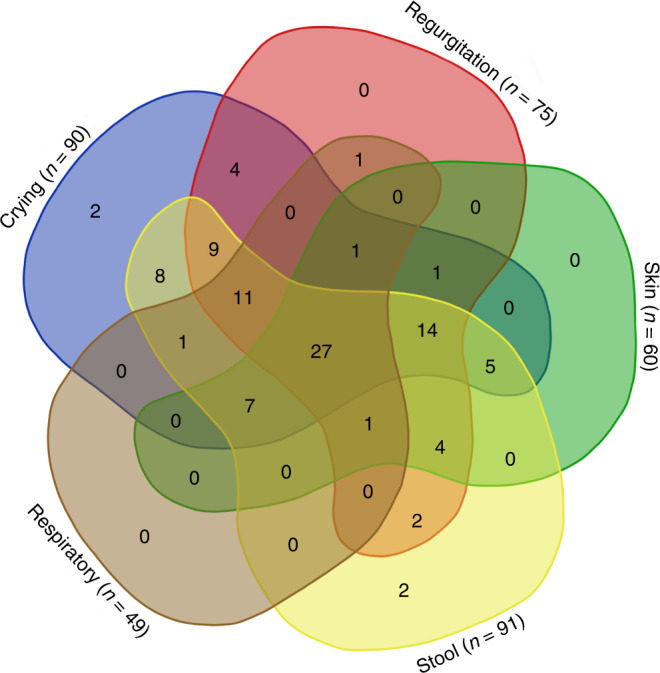
Venn diagram for CoMiSS symptoms at presentation (T0). Venn diagram illustrates the relationships between items of CoMiSS that overlap (overlapping areas) or do not overlap (stand-alone). For example: Stool changes includes all children who reported stool changes (as the most prevalent manifestations) even if they overlapped with other areas. By summing all the yellow values, we can determine that the number of children with stool changes was 91, followed by crying 90, regurgitation 75, skin manifestations 60 and, finally, respiratory manifestations 49.

Stool changes according to BSS were the most frequently reported symptom of CoMiSS (91% of all cases), which includes diarrhea (types 7 and 6) in 77% of cases (80.9% versus 56.25%), constipation (types 1 and 2) in 9% (8.3 versus 12.5%) and soft stool (type 5) in 5% (4.7 versus 6.25%) in the confirmed CMA group versus negative CMA group, respectively, then crying and regurgitation in 90 and 75% of all cases, respectively. Details of items of CoMiSS at presentation (T0) are illustrated in Fig. 1 [Fig. 1: Venn diagram illustrates the relationships between items of CoMiSS which overlap (overlapping areas) or don’t overlap (stand-alone). For example: crying (the Violet Blue area) includes all children who reported crying even if they overlapped with other areas. By summing all the blue values, we can determine that the number of crying children was 90].

There was a statistically significant difference between the confirmed CMA and CMA negative groups regarding the presence of stool changes and skin manifestations at T0. Table 3 illustrates symptoms included in CoMiSS between confirmed CMA and negative CMA groups at T0. It’s noteworthy to note that patients also reported one or more symptoms, some of which were not specified in CoMiSS and others were.

**Table 3:** Comparison of symptoms included in CoMiSS between the CMA confirmed and negative groups at T0.

	Crying			Regurgitation			Stool			Skin			Respiratory		
	Cow milk allergy		*p*	Cow milk allergy		*p*	Cow milk allergy		*p*	Cow milk allergy		*p*	Cow milk allergy		*p*
Group	Negative *N* (%)	Confirmed *N* (%)		Negative *N* (%)	Confirmed *N* (%)		Negative *N* (%)	Confirmed *N* (%)		Negative *N* (%)	Confirmed *N* (%)		Negative *N* (%)	Confirmed *N* (%)	
Yes	13 (81.25%)	77 (91.67%)	0.204 NS	12 (75.00%)	63 (75.00%)	1.000 NS	12 (75.00%)	79 (94.05%)	0.015*	6 (37.50%)	54 (64.29%)	0.046*	7 (43.75%)	42 (50.00%)	0.647 NS
No	3 (18.75%)	7 (8.33%)		4 (25.00%)	21 (25.00%)		4 (25.00%)	5 (5.95%)		10 (62.50%)	30 (35.71%)		9 (56.25%)	42 (50.00%)	
Total score	16 (100.00%)	84 (100.00%)		16 (100.00%)	84 (100.00%)		16 (100.00%)	84 (100.00%)		16 (100.00%)	84 (100.00%)		16 (100.00%)	84 (100.00%)	
0	3 (18.75%)	7 (8.33%)	0.204 NS	4 (25.00%)	21 (25.00%)	1.00 NS	4 (25.00%)	5 (5.95%)	0.015*	10 (62.50%)	30 (35.71%)	0.046*	9 (56.25%)	42 (50.00%)	0.646 NS
1	3 (18.75%)	7 (8.33%)	0.204 NS	1 (6.25%)	4 (4.76%)	0.802 NS	NA	NA	NA	0 (0.00%)	3 (3.57%)	0.441 NS	2 (12.50%)	15 (17.86%)	0.603 NS
2	1 (6.25%)	3 (3.57%)	0.617 NS	1 (6.25%)	5 (5.95%)	0.960 NS	1 (6.25%)	4 (4.76%)	0.803 NS	4 (25.00%)	21 (25.00%)	1.00 NS	3 (18.75%)	12 (14.29%)	0.646 NS
3	0 (0.00%)	5 (5.96%)	0.317 NS	1 (6.25%)	3 (3.57%)	0.617 NS	NA	NA	NA	2 (12.50%)	14 (16.67%)	0.674 NS	2 (12.50%)	15 (17.86%)	0.603 NS
4	3 (18.75%)	3 (3.57%)	0.019*	1 (6.25%)	6 (7.14%)	0.896 NS	4 (25.00%)	19 (22.62%)	0.834 NS	0 (0.00%)	3 (3.57%)	0.441 NS	NA	NA	NA
5	0 (0.00%)	0 (0.00%)	NA	0 (0.00%)	8 (9.52%)	0.197 NS	NA	NA	NA	0 (0.00%)	4 (4.76%)	0.373 NS	NA	NA	NA
6	6 (37.50%)	59 (70.24%)	0.012*	8 (50.00%)	37 (44.05%)	0.660 NS	7 (43.75%)	56 (66.67%)	0.081 NS	0 (0.00%)	9 (10.71%)	0.0171 NS	NA	NA	NA

*N* number of infants, *YES* the symptom was reported, * statistically significant, *NS* non significant.

*NA* not applicable because CoMiSS score does not include this point score for this item in the stool or in respiratory symptoms.

Stool change items include diarrhea, soft stool, normal stool, and constipation.

Score 4 in stool changes refers to both constipation (2 cases in negative CMA group and 7 cases in confirmed CMA group) and diarrhea (2 cases in the negative CMA group, 12 cases in the confirmed CMA group).

### CoMiSS evaluation

**Fig. 2:**
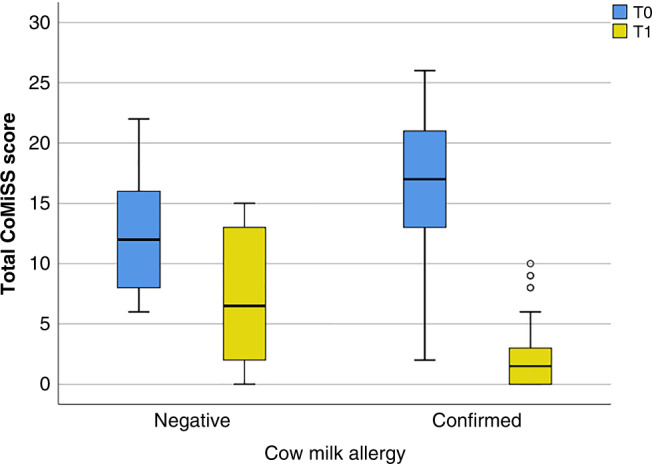
Comparison between CMA confirmed and negative group regarding CoMiSS score at T0 and T1. According to this figure, the CoMiSS score before elimination for the negative group ranges from 6.00 to 22.00 with a median of 12.00, whereas for the confirmed group, the range is 2.00-26.00 with a median of 17.00. After 1 month of elimination, the negative group’s CoMiSS score ranges from 0.00 to 15.00 with a median of 6.50, whereas the confirmed group’s score ranges from 0.00 to 10.00 with a median of 1.50.

The confirmed CMA group’s median CoMiSS score at presentation (T0) was considerably higher than the group with negative CMA results (median 17 versus 12, range 2.00–26.00 versus 6.00–22.00). The median IQR of all CoMiSS parameters, except for the regurgitation and respiratory symptoms scores, was significantly higher in the confirmed CMA group at T0. Following CMFD (T1), 82/84 (97.62%) of the confirmed CMA group reported a significant reduction in total CoMiSS, compared to 8/16 (50%) of the negative CMA group (median 1.5 in the confirmed CMA group versus 6.5 in the CMA negative group). Additionally, a statistically significant difference was seen in the median score and percentage improvement of crying, regurgitation, stool changes and skin symptoms, as well as in the percentage improvement of CoMiSS following CMFD (−91.41% versus −36.11%). Comparison of CoMiSS score initially (T0) and after 4 weeks of CMFD (T1) in both groups is shown in Table 4 and Fig. 2.

**Table 4:** CoMiSS score initially (T0) and after 4 weeks of CMFD (T1) in both the groups.

		Negative CMA (*n* = 16)	Confirmed CMA (*n* = 84)	Test of significance, *p* value
*Total CoMiSS Score T0*				
Median [min–max]		12.00 [6.00–22.00]	17.00 [2.00–26.00]	0.006*
Mean ± SD		12.44 ± 5.01	16.39 ± 5.13	
Crying: median [IQR]		4.00 [5.00]	6.00 [3.00]	0.016*
Regurgitation: median [IQR]		5.00 [5.50]	5.00 [1.00]	0.905 NS
Stool: median [IQR]		4.00 [5.00]	6.00 [2.00]	0.038*
Skin: median [IQR]		0.00 [2.00]	2.00 [6.00]	0.026*
Respiratory: median [IQR]		0.00 [2.00]	0.50 [2.00]	0.669 NS
*Total CoMiSS Score T1*				
Median [min–max]		6.50 [0.00–15.00]	1.50 [0.00–10.00]	0.001*
Mean ± SD		7.19 ± 5.55	2.07 ± 2.24	
Crying: median [IQR]		1.50 [4.00]	0.00 [2.00]	0.040*
Regurgitation: median [IQR]		1.50 [3.00]	0.00 [0.00]	<0.0001*
Stool: median [IQR]]		0.00 [5.00]	0.00 [0.00]	0.002*
Skin: median [IQR]		0.00 [1.50]	0.00 [0.00]	0.037*
Respiratory: median [IQR]		0.00 [0.00]	0.00 [1.00]	0.598 NS
*Total CoMiSS Score percentage change*				
Median [IQR]		−36.11 [−78.81]	−91.41 [−18.35]	<0.0001*
Mean ± SD		−44.33 ± 40.47	−87.22 ± 13.97	
*Percentage of patients improved after CMFD (n, %)*				
Crying	78/90 (86.67%)	5/13 (38.46%)	73/77 (94.81%)	<0.0001*
Regurgitation	69/75 (92.00%)	8/12 (66.67%)	61/63 (96.83%)	0.0004*
Stool	82/91 (90.11%)	6/12 (50.00%)	76/79 (96.20%)	<0.0001*
Skin	54/60 (90.00%)	2/6 (33.33%)	52/54 (96.30%)	<0.0001*
Respiratory	34/49 (69.39%)	5/7 (71.43%)	29/42 (69.05%)	0.429 NS
*Total CoMiSS Score percentage change*				
>50% reduction in total score		8 (50.00%)	82 (97.62%)	<0.0001*

*IQR* interquartile range, * statistically significant, *NS* non significant.

### ROC curve

**Fig. 3:**
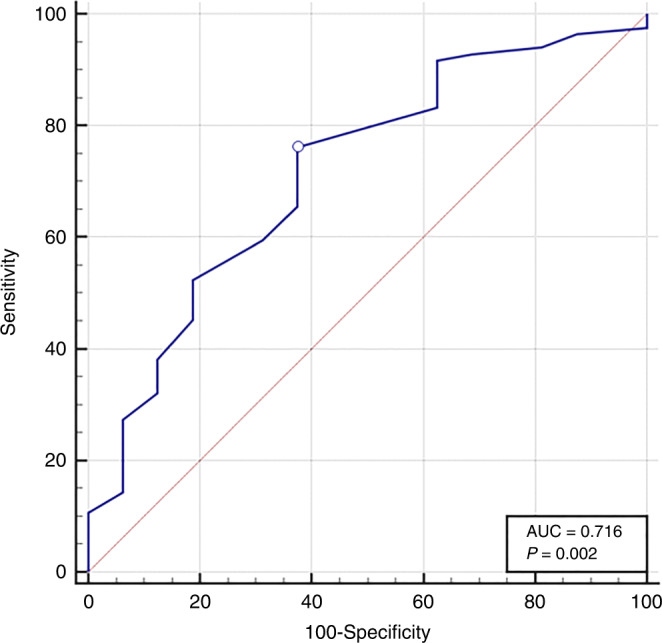
ROC curve of the CoMiSS score. Identified the score of ≥12 as the best cut-off point suspecting CMA diagnosis (area under the curve 0.716) with a sensitivity of 76.19%, specificity of 62.50%, PPV of 91.43, NPV of 33.33, and overall accuracy of 74.00%. In contrast, cut-off point of ≥10 shows 84.52% sensitivity, 37.50% specificity, 87.7% PPV, and 31.6% NPV.

The ROC curve identified the score of ≥12 as the best cut-off point (area under the curve 0.716) with a sensitivity of 76.19%, specificity of 62.50%, PPV of 91.43, NPV of 33.33, and overall accuracy of 74.00%. In contrast, cut-off point of ≥10 shows 84.52% sensitivity, 37.50% specificity, 87.7% PPV, and 31.6% NPV (Fig. 3).

### Manifestations other than CoMiSS

At presentation (T0), 80% of infants had a persistent mucoid stool,41% had a bloody stool and 52% were faltering growth. Significantly more mucoid stools were reported in the confirmed CMA group than the negative group. Following CMFD (T1), there was a statistically significant difference between the confirmed CMA group and the negative group in terms of improvements in mucoid stool (93.06% versus 62.50%, *p* value 0.006%), bloody stool (94.59% versus 50%, *p* value 0.004%), abdominal distention not resolving on regular treatment (90.63% versus 61.54%, *p* value 0.006%), and weight gain (median of 500.00 g versus 225 g, *p* value 0.001%). Table 5 details manifestations not included in CoMiSS and their improvement following CMFD.

**Table 5:** Manifestations not included in CoMiSS score and their improvement in response to CMFD.

	All patients (*n* = 100)	Cow milk allergy		Test of significance *p* value
		Negative (*n* = 16)	Confirmed (*n* = 84)	
Persistent significant mucoid stool	80/100 (80.00%)	8/16 (50.00%)	72/84 (85.71%)	0.001*
Patients with improved mucoid stool (out of all patients with positive mucoid before (*n* = 80))	72/80 (90.00%)	5/8 (62.50%)	67/72 (93.06%)	0.006*
Bloody stool	41/100 (41.00%)	4/16 4 (25.00%)	37/84 (44.05%)	0.155 NS
Patients with improved bloody stool (out of all patients with bloody stool before (*n* = 41))	37/41 (90.24%)	2/4 (50.00%)	35/37 (94.59%)	0.004*
Hematemesis	11/100 (11.00%)	2/16 (12.50%)	9/84 (10.71%)	0.834 NS
Patients with improved hematemesis (out of all patients with hematemesis before (*n* = 11))	10/11 (90.90%)	2/2 (100.00%)	8/9 (88.89%)	0.624 NS
Marked abdominal distention not resolving on regular treatment	77/100 (77.00%)	13/16 (81.25%)	64/84 (76.19%)	0.659 NS
Patients with improved abdominal distention (out of all patients with abdominal distention before (*n* = 77))	66/77 85.71%	8/13 (61.54%)	58/64 (90.63%)	0.006*
Faltering growth	52/100 (52.00%)	10/16 (62.50%)	42/84 (50.00%)	0.421 NS
Weight gain after 1 month of elimination (g), median [IQR]	470 [250]	225 [300]	500 [225]	0.001*

* statistically significant, *NS* non significant.

## Discussion

CMA diagnosis is challenging as there is no specific clinical presentation suggestive of CMA diagnosis. In addition, symptoms and signs of CMA may overlap with functional GI disorders (colic, regurgitation, and constipation) and OFC is the only gold standard method for CMA diagnosis.^7^ In our study population of 100 infants with persistent symptoms suggestive of CMA, 84% of them were diagnosed confirmed CMA by OFC.

Gastrointestinal symptoms were the most common presenting symptoms either alone in 25% of infants or with other system affection as 24% presented with GI and skin manifestations,13% with GI and respiratory manifestations and 36% presented with GI, respiratory and skin manifestations. Skin and respiratory tract manifestations occur frequently in infants with CMA and when GI and/or general manifestations are combined with skin and/or respiratory tract manifestations, the presence of CMA is more likely.^17,18^

Analysis of CoMiSS GI manifestations in confirmed CMA group revealed that diarrhea was the most frequently reported symptom in 80.9%, followed by regurgitation in 75% then constipation in 8.3%. These percentages when comparable with another study in our country which reported the prevalence of gastrointestinal GI manifestations associated with CMA as regurgitation 92%, diarrhea 80%, colic 75% and lastly constipation 5%.^19^

Median CoMiSS at presentation (T0) was ≥12, with a higher median initial score (T0) in the confirmed CMA group 17.00 [min-max 2.00-26.00] than in the CMA negative group 12.00 [min-max 6.00-22.00]. CoMiSS is considered a useful awareness tool for CM-related symptoms and a cut-off point of 12 was suggested as a possible score to indicate CMA with a good, reported correlation between positive CoMiSS (≥12) and a positive response to CMFD. Recently, the previously proposed cut-off point which indicates the likelihood that symptoms may be cow’s milk related was suggested to be lowered from *≥*12 to *≥*10.^9^

The best cut-off value was different in multiple studies.The discrepancy in cut-off values, ranging from ≥5.5 to ≥12, can be explained by the differences in study design: while some studies used a CoMiSS above a specific cut-off as an inclusion criterion,^7,8^ other studies used symptoms as an inclusion criterion and determined CoMiSS as additional information.^10,11,19–22^ The range of values suggests that CoMiSS may operate differently according to study design and type of symptoms presented.

Eleven studies documented that a score of ≥12 is predictive of a favorable response to a CMFD, showing an estimated sensitivity between 20% and 77%, a specificity of 54% to 92% for the diagnosis of CMA. In contrast, the sensitivity of 20% for the cut-off ≥12 was reported in a study including mainly infants with hematochezia.^2^

In our study, the ROC curve identified the cut-off point of ≥12 as the best diagnostic cut-off score with a sensitivity of 76.19%, specificity of 62.50%, PPV of 91.43%, NPV of 33.33%, and Overall Accuracy of 74.00%. In contrast, cut-off point of ≥10 shows 84.52% sensitivity, 37.50% specificity, 87.7% PPV and 31.6% NPV. In our study CoMiSS was not only a good tool for prediction of patients who would benefit from CMFD, but also was beneficial in avoiding CMA underdiagnosis which was prevalent in our patients especially in patients with more than one system affection.

We thought that our median of CoMiSS score was higher than some studies as crying (considered when reported for one week or more without any obvious cause) was prevalent in 90% of our cases and had a major part of the score than any other parameter (65% of our infants had a score of 6). Parental perception of severity and duration of crying is subjective and may be over-reported. So, measuring crying with objective tools and diary recording may result in more exact data.^9^

In our study, Infants with any symptoms suggestive of CMA (except for clear immediate IgE reactions as anaphylaxis, urticaria and angioedema) were included, regardless of initial CoMiSS at presentation (T0) and including infants with both IgE positive and negative test results. In fact, CoMiSS does contain symptoms which are IgE mediated, such as urticaria but also vomiting, diarrhea and other gastrointestinal (GI) symptoms that can be either IgE as non-IgE mediated.^9^

Following CMFD for 4 weeks in our study, CoMiSS (T1) decrease was more pronounced in confirmed CMA group than in the negative group with a significant response occurred in 97.62% of confirmed CMA group. Twelve reports had a significant reduction in CoMiSS after an elimination diet.^23^ Moreover, a reduction of >50% was predictive of a subsequent positive OFC.^9^

According to our findings, the median values for stool changes, skin symptoms, and crying were significantly different between the infants with confirmed CMA and those with negative CMA at presentation. Additionally, after 4 weeks on CMFD, the confirmed CMA group showed significant improvement than the negative group in terms of crying, stool changes, regurgitation, and skin symptoms regarding median value and percentage improvement. However, respiratory parameter did not show significant difference between both groups either before or after CMFD.

In our study, respiratory manifestations occurred frequently (in 50% of confirmed CMA group), this high frequency can be explained as infants with an atopy predisposition have delayed maturation of the T helper 1 response which puts them at higher risk for infection in the first few years of life. Additionally, a recent retrospective study revealed that in infants under the age of two, sensitization to β-lactoglobulin (a protein found in cow’s milk) was linked to a nearly four-fold greater risk of recurrent respiratory tract infections.^18^

The inclusion of respiratory symptoms in the CoMiSS has been debated since the vast majority of respiratory symptoms in infants are caused by (viral) infections. Nevertheless, respiratory symptoms are also listed by major guidelines as being possibly related to CMA.^24^ CoMiSS mentions that the symptoms should be “chronic, and not related to infection”. Distinguishing between respiratory symptoms caused by infection or allergy remains challenging during infancy.^9^

It is suggested that CMA should be considered as a cause of persistent mucoid stool, bloody stool, hematemesis, abdominal distention not resolving on regular symptomatic treatment and slow weight gain.^25,26^ Being not included in CoMiSS, these findings usually associate with low CoMiSS score.

In all, 52% of our patients were underweight at presentation, this high rate can be explained by underdiagnosis of CMA as 57% of our patients were misdiagnosed as functional GI disorders and underwent frequent change of milk formulas (Lactose-free formula (LF), anti-regurgitation (AR) formula, Soy-based formula or partially hydrolyzed formula (HA). Faltering growth is an alarming symptom requiring referral and a broad diagnostic work-up for full understanding of the cause. Since CoMiSS is an awareness tool and multiple factors and underlying disease may determine faltering growth, this was not included in CoMiSS.^9^ Despite these diagnostic challenges, a timely diagnosis of CMA is crucial to improve faltering growth and quality of life, which may persist even despite effective management.^27^

Food protein-induced allergic proctocolitis (FPIAP) often presents as rectal bleeding, hematochezia, or persistent mucus-streaked diarrhea in an otherwise healthy young infant.^28^ FPIAP prevalence estimates range widely from 0.16% in healthy children to 64% in infants with blood in stools.^29^ This disease usually manifests within the first weeks of life and resolves by late infancy in most cases.

Our results revealed a significant difference regarding persistent mucoid stool before elimination (T0) with significant improvement of mucoid stool, bloody stool, abdominal distention, and weight gain in response to CMFD in confirmed CMA group, emphasizing the importance of systematic evaluation and objective scoring of these symptoms to improve CoMiSS accuracy as a useful screening tool in CMA diagnosis.

Our study’s strength was that it included all infants with symptoms suggestive of CMA, with no cut-off value for CoMiSS, we started medical treatment initially to minimize CMA overdiagnosis, then CMFD was started in individuals with persistent symptoms, CoMiSS scoring was performed by the same pediatric gastroenterologist before and after CMFD. Also, we included other presenting symptoms that were possibly related to CMA as rectal bleeding, persistent mucoid stool, hematemesis, abdominal distension, and Faltering growth, then we monitored their improvement in response to CMFD. In addition, all infants had OFC to confirm CMA diagnosis and to rule out those whose symptoms improved over time. Infants whose parents declined OFC for fear of the return of symptoms were excluded from the study.

## Limitation of the study

The major limitation of our study was that 30 infants were excluded from the study because their mothers were non-adherent to CMFD, refused OFC, or were lost during follow-up. Moreover, most of our infants scored highly on crying as reported by their parents in our study.

## Conclusions

CoMiSS is a good tool for identifying infants who may benefit from CMFD. Our study revealed a CoMiSS score of ≥12 to be the best cut-off point. However, CoMiSS cannot be used alone for accurate diagnosis of CMA. So, we recommend combining CoMiSS improvement in response to CMFD with the initial CoMiSS score and including other symptoms commonly associated with CMA as Mucoid stool, bloody stool, marked abdominal distention not responding to medical treatment, and faltering growth to CoMiSS to improve CoMiSS score accuracy in CMA diagnosis. However, more research is needed for a full evaluation.

## Data Availability

All datasets presented in this study are included in the article.
